# Do Laboratory Mouse Females that Lose Their Litters Behave Differently around Parturition?

**DOI:** 10.1371/journal.pone.0161238

**Published:** 2016-08-30

**Authors:** Elin M. Weber, Jan Hultgren, Bo Algers, I. Anna S. Olsson

**Affiliations:** 1 Department of Animal Environment and Health, Swedish University of Agricultural Sciences, Skara, Sweden; 2 Department of Comparative Medicine, Stanford University, Stanford, California, United States of America; 3 i3S—Instituto de Investigação e Inovação em Saúde, Universidade do Porto, Porto, Portugal; 4 Laboratory Animal Science, IBMC-Instituto de Biologia Molecular e Celular, Universidade do Porto, Porto, Portugal; Harvard University Faculty of Arts and Sciences, UNITED STATES

## Abstract

Efficiency in laboratory mouse breeding is hampered by poor reproductive performance, including the loss of entire litters shortly after birth. However, the underlying mechanisms are not yet fully understood and establishing the cause of death in laboratory mouse pups can be complicated. Newborn mouse pups are generally hidden in nests, dead pups are often eaten by the female, and the widespread practice of leaving periparturient females undisturbed complicates inspection, which may delay the discovery of pup loss. In order to efficiently prevent problems with litter loss, it is important to find key factors for survival. We investigated differences in periparturient behavior between female laboratory mice whose pups survived until weaning and females whose entire litters were lost. Video recordings of 82 primiparous females of the C57BL/6 strain or knockouts with C57BL/6 background were used. The mice were observed from 24 h before until 24 h after parturition and female behaviors coded using a pre-established ethogram. The relationship between behavior and survival was analyzed using logistic models, where litter survival was regressed on the proportion of 30-s observations with at least one occurrence of the behavior. We found that females with surviving litters performed more nest building behavior during the last 24 h before parturition (p = 0.004) and spent less time outside the nest during the entire observation period (p = 0.001). Increased litter survival was also associated with more passive maternal behaviors and the female ignoring still pups less. Females that lost their litters performed more parturition-related behaviors, suggesting prolonged labor. The results indicate that maternal behavior plays a significant role in laboratory mouse pup survival. Complications at parturition also contribute to litter mortality.

## 1. Introduction

The mouse is the predominant mammal species used as a model organism in research, representing more than half of all animals used for experimental purposes in Europe [1]. With a great number of genetic modifications, an ordinary laboratory animal facility may keep hundreds of breeding colonies of mice with different genetic backgrounds. Breeding efficiency is often hampered by problems with reproduction, including pre-weaning pup mortality. In a previous study [2] we found a total mortality rate (calculated as percentage of entire litters being lost before weaning at around 21 days) of 32% for C57BL/6 and 20% for BALB/c, two of the most common strains of laboratory mice. However, reported mortality rates vary greatly; from nearly 0 to 50% in scientific studies of C57BL/6 mice [3–5] compared to 13% reported by a commercial breeder [6].

Establishing the cause of death in laboratory mouse pups is complicated for a number of reasons. Firstly, newborn pups are small and hidden in nests and dead pups are often eaten by the female. Moreover, there is a widespread practice of leaving periparturient mouse females undisturbed, which may delay or prevent the discovery of pup loss. In farm animals, perinatal mortality is relatively well studied and the major causes of death are similar across species, namely hypothermia, maternal underfeeding, inappropriate maternal behavior, infections and injuries [7]. Although there is experimental evidence that a poor maternal diet [8] and maternal infection [9] increase infant mortality also in mice, these factors are likely to be less important in normal breeding where diets are balanced and housing is microbiologically controlled. On the other hand, mouse pups are born without fur and therefore very sensitive to hypothermia, and a recent study done under commercial breeding conditions showed that a 27% reduction in pup mortality can be achieved by providing C57BL/6 mice with nesting material [10].

The observed high mortality in several transgenic and knockout mice is often attributed to impaired maternal behavior [11] but many studies are designed in such a way that an influence of poor pup health cannot be ruled out [12], and the two factors (maternal behavior and pup health) may also interact: stimuli provided by moving pups are crucial for the female to maintain maternal care [11]. Several researchers have investigated the effect of environmental factors on reproductive performance in mice [3, 13–18]. However, even though mouse pups are totally dependent on their mother for survival, few studies have investigated the effect of maternal behavior on pup survival. Brown *et al*. [19] found strain differences in both maternal behavior and litter survival when comparing two strains of mice, but no comparisons were made between females that successfully raised their litters and females whose litters died. The authors reported that the reason why pups died was unknown. High numbers of newborn dying shortly after birth have also been reported in farmed mink [20] and pigs [21]. Malmkvist *et al*. [22] observed female mink around and during parturition and found several behavioral differences between females who successfully raised a litter, and females with a high proportion of kits dying. Prolonged parturition has also been reported to increase neonatal mortality in pigs and mink [22, 23].

It has been speculated that infanticide, the most extreme form of inappropriate maternal behavior, is an important cause of perinatal death in mice (*e*.*g*. [18]). Whereas dead pups are often cannibalised by their parents, only two studies have described injuries inflicted by parents to be a likely cause of death [24, 25]. Using detailed behavioral observations of females losing their litters within the first three days after birth, we have not found any evidence that C57BL/6 females actively kill their pups. In most cases, pups that later died were active after birth and displayed successively fewer movements until they were finally lying still [26].

The present study aimed to investigate differences in periparturient behavior between female laboratory mice whose pups survived until weaning and females whose entire litters were lost. We hypothesized that maternal behavior would differ between these females, and focused the observations on important aspects of maternal behavior such as nest building behavior, different aspects of pup-related behaviors and time the female spent inside and outside the nest.

## 2. Material and Methods

### 2.1. Video material

We used video recordings collected during two experiments conducted between June and September 2005 (recording A) and between August 2006 and March 2007 (recording B) at the Institute for Molecular and Cell Biology, Porto, Portugal. The experiments were carried out under a project license (ref. 003758) issued by the Direcção Geral de Veterinária, the competent authority for animal protection in Portugal, and focused on maternal behavior and reproduction in C57BL/6, the most commonly used mouse strain in biomedical research, and also development of iron load in the knockouts *Hfe-/-* and *β2m-/-* (for more information on these knockouts, see [27, 28]).

A total of 82 primiparous mice were used. They were either C57BL/6 mice from a local breeding colony originally sourced from Harlan Interfauna Iberica (Barcelona, Spain) (recording A, n = 20; recording B, n = 20) or knockouts *Hfe*-/- (recording B, n = 22) and *β2m-/-* (recording B, n = 20) with a C57BL/6 background. The mice were kept in four different housing systems (Table 1). Females from recording A were mated in trios (one male and two females) and after separation from the males they were placed in either standard polycarbonate Makrolon II cages (Tecniplast, Italy) provided with corncob bedding (n = 10) or Makrolon III cages (Tecniplast, Italy) provided with corncob bedding, 100 ml of aspen bedding, a chew block, half a sheet of tissue paper, a translucent red PVC nest box (MouseHouse, Tecniplast, Italy) and a cardboard nest box (Des Res., Lillico Biotechnology, UK) (n = 10). Females in recording B were housed from mating together with the male in Makrolon II cages with corncob bedding and half a nestlet (Lillico Biotechnology, UK) per cage (n = 31), or one nestlet, a chew block, a transparent tinted polycarbonate mouse tunnel (Datesand Ltd, UK) hanging from the grid and a cardboard tube cut to provide as a nest box (Datesand Ltd, UK) (n = 31). In order to synchronise oestrus cycles [14] nest material from the males’ home cages was placed in the females’ cages at 3 days before mating. Approximately 14 days after mating, the males were removed and females were housed singly. Room temperature was kept at 19–23°C and relative humidity at 65–72% in both recordings. The animals were maintained on a 12-h light: 12-h dark cycle with lights on at 05:00. They were given standard feed (Mucedola RF25, Mucedola, Italy) and autoclaved tap water *ad libitum*. Day of birth was determined by daily visual inspections. Cages were cleaned once a week, except after parturition when the females were left undisturbed until day 10 (recording A) or 4 (recording B). In each cage the pups were counted at first cage cleaning after parturition. If dead pups were found during daily visual inspections before cage cleaning (*i*.*e*. without opening the cages), they were removed from the cage.

**Table 1:** Housing systems, mouse genotype and number of females studied (see also S1 Fig).

Recording	Cage type, LxWxH (mm)	Bedding and nesting material	Furnishment	Mouse genotype	Number of females
A	Makrolon II (265×205×140)	Corncob; no nesting material	None	C57BL/6	10
	Makrolon III (265 x 410x175)	Corncob, aspen bedding; half a tissue paper	Modified cardboard nest box, translucent red PVC nest box, chew block	C57BL/6	10
B	Makrolon II (265×205×140)	Corncob; 0.5 Nestlet	None	C57BL/6	10
				*Hfe-/-*	11
				*β2m-/-*	10
	Makrolon II (265×205×140)	Corncob; 1 Nestlet	Modified cardboard tube (as nest box), transparent tinted polycarbonate mouse tunnel, chew block	C57BL/6	10
				*Hfe-/-*	11
				*β2m-/-*	10

The animals were video-recorded in their home cages before and after parturition. Four cages were recorded simultaneously using cameras (Ikegami ICD-47E, B/W CCD, Japan) connected to a time lapse recorder (Panasonic AG-TL750E, Thailand). The recordings were rotated by means of a camera switcher (Sanyo VQC 809-P, Japan) at 30-s intervals, and each cage was thus in view for totally 15 min per hour. During dark hours, infrared lights (Monacor, P 1204ST, Sweden) were used. In recording B, approximately one third of the cages were recorded continuously throughout the recording period and data were collected into a computer with a multi-camera vigilance system (GV-800/8; GeoVision, Taiwan).

### 2.2 Data collection

Data were collected from the video recordings. To determine the exact time when parturition began, the recordings were scanned. After detection of pups the film was rewound and played at fast forward to find the female in birth position [29]. Time of parturition was defined as the time when the first pup was delivered, or (if the pup was not seen) the first time when the female was seen in birth position. If neither the first pup nor birth position was possible to detect, time for parturition was estimated as the midpoint between the last time the female was seen pregnant and the first time a pup was seen or the female was seen non-pregnant.

Litter survival was defined as at least one pup surviving until weaning at day 21 after birth. Female behavior was observed during a 30-s period every 15 min from 24 h before until 24 h after parturition and coded by one observer using a predefined ethogram (see S1 Ethogram). The behaviors were grouped into six categories, relating to the behavior or location of the female (Table 2). Of the 82 females that were mated, 78 females were pregnant and gave birth to a litter. Females with more than 12 h of video recordings missing or of insufficient quality were excluded from the analysis. At the start of behavioral observations, the observer was blinded to the survival of the litters.

**Table 2:** Overview of the studied female behaviors included in each behavior category. For a detailed definition of the behaviors, see S1 Ethogram.

Behavior category	Behaviors included
Nest building	Nest building, Moving nest
Parturition-related	Giving birth, In labor position, Dystocia
Active maternal behavior	Being active in nest, Being active with pup, Retrieving still pup, Retrieving moving pup, Carrying pup, Moving pup
Passive maternal behavior	Nursing, Being still in nest
Self-oriented	Resting alone, Resting outside nest, Ignoring moving pup, Ignoring still pup, Self-grooming, Hunched posture, Digging, Stretching, Eating
Abnormal	Removing pup, Eating pup, Chasing own tail, Gnawing bars, Climbing bars, Other abnormal

### 2.3. Statistical analysis

Basic descriptive statistics were prepared in Excel 2010 (Microsoft Corp., Redmond, WA, USA) and further analyses were made in Stata/IC 13 (StataCorp SLP, College Station, Texas, USA). Data were arranged in long format with one 30-s observation per 15 min. The observation period was divided into forty-eight 1-h, sixteen 3-h, eight 6-h, and two 24-h sub-periods, and data were averaged by sub-period, as well as for the entire observation period, by calculating the proportion of observations with at least one occurrence of the behavior. Similarly, aggregated values for the six behavior categories (Table 2) were calculated as the proportion of observations with at least one occurrence of any of the behaviors in a category. For each behavior and behavior category, the mean proportions for different sub-periods were plotted against time, separately for females with litters having survived and not having survived.

Associations between female behavior and litter survival were analyzed in three steps. In the first step, univariable logistic models of survival (0 = all pups died; 1 = at least one pup survived) were constructed using the logit command in Stata, each one containing one of ten single or aggregated behavior variables representing (1) nest building before parturition, (2) parturition-related behavior during the last 6 h before parturition, (3) passive and (4) active maternal behavior after parturition, (5) nursing after parturition, (6) self-oriented behavior after parturition, (7) ignoring still and (8) ignoring moving pup after parturition, (9) being outside nest during the whole observation period, and (10) abnormal behavior during the whole observation period. All behavior traits were treated as continuous variables. Square and cubic terms were tested to allow for curvilinear relationships. Effects with p≤0.05 were regarded as behavior variables eligible for subsequent analysis.

In the second step, the effect of potentially confounding factors was examined. Litter survival was regressed on each one of three variables representing recording (A or B), cage design (furnished or not), and mouse genotype (wildtype C57BL/6, *Hfe-/-* or *β2m-/-*) in univariable logistic models. Effects with p≤0.25 were regarded as potentially confounding factors to be included in subsequent analysis. In the third analytical step, eligible behavior variables and potentially confounding factors were used to construct a multivariable logistic regression model of survival. All possible combinations of behavior variables were tested for inclusion together with potential confounders, and the model with the lowest Bayesian Information Criteria (BIC) value was accepted as the best model. Finally, plausible two-way interactions between the main effects were tested and retained if p≤0.05.

The final multivariable model was validated by Pearson Chi-square and Hosmer-Lemeshow goodness-of-fit tests, and by examining standardised Pearson residuals. Predictive marginal means were calculated and plotted with 95% confidence intervals against different values of the covariates.

## 3. Results

**Fig 1:**
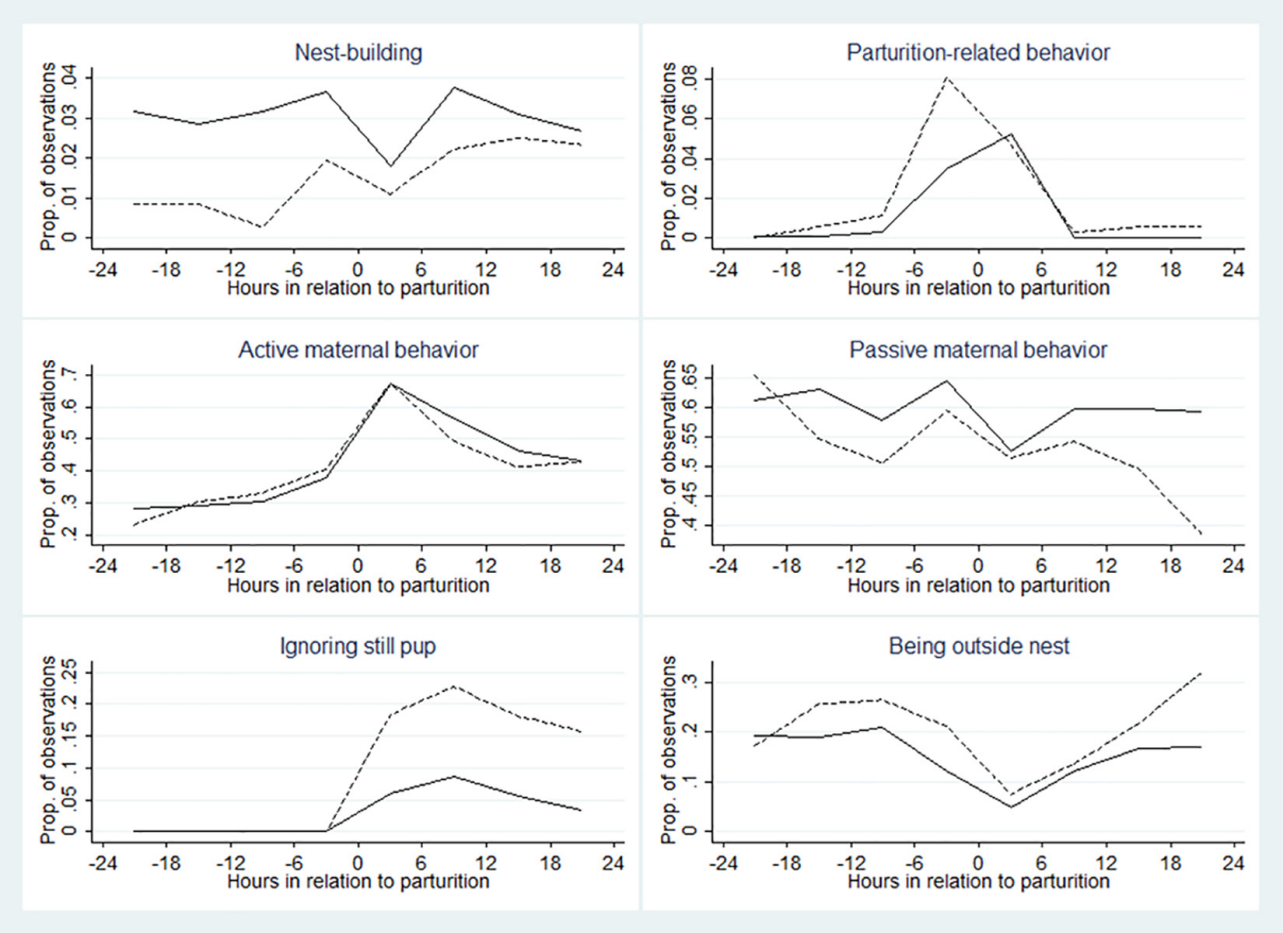
Observed relationship between female laboratory mouse behaviors and litter survival. Nest building, parturition-related behavior, active and passive maternal behavior, ignoring still pup and being outside nest from 24 h before until 24 h after parturition in 64 primiparous mice whose litters survived (solid line; n = 49) and did not survive until weaning (dashed line; n = 15); mean proportion of 30-s observations per 6-h period with at least one occurrence of the behavior.

Data from 64 females (recording A, n = 17; recording B, n = 47) could be analyzed, of which 49 females produced a weaned litter (overall 77% survival). In 14 females (13 surviving litters and 1 lost), more than 12 h of recordings were missing or of insufficient quality and these females were therefore excluded. All data are available in S1 Data. We found significant associations of litter survival with 5 of the 10 female behaviors tested (Table 3 and Fig 1). Females that successfully weaned a litter showed more nest building behavior during the last 24 h before parturition (p = 0.004) and survival of the litter was associated with the female being less outside the nest between 24 h before and 24 h after parturition (p = 0.001). Increased litter survival was also associated with more passive maternal behaviors being performed (p = 0.006) and the female ignoring still pups less during the first 24 h after parturition (p = 0.035). Females that lost their litters performed more parturition-related behaviors during the last 6 h before giving birth (p = 0.020). The probability of survival of litters also followed a curvilinear relationship with active maternal behavior during the first 24 h after birth, with a maximum probability of survival around a mean behavior frequency of 0.55 (not in table). No significant effect on litter survival could be shown of nursing, self-oriented behavior, abnormal behavior, or ignoring moving pup.

**Table 3:** Summary of five univariable logistic models of litter survival. The table gives an overview of the 64 primiparous laboratory mice, in which significant (p≤0.05) linear associations with behaviors or behavior categories were found.

Behavior variable^1^	Coefficent	Standard. error	OR^2^	p
Nest building before parturition	76.7	26.5	2.2	0.004
Parturition-related behavior during last 6 h before parturition	-11.9	5.13	0.89	0.020
Passive maternal behavior after parturition	9.60	3.52	1.1	0.006
Ignoring still pup after parturition	-3.56	1.69	0.96	0.035
Being outside nest during observation period	-26.6	7.98	0.77	<0.001

^1^ Proportion of 30-s observations with at least one occurrence of the behavior.

^2^ OR = change in odds of litter survival per percent unit increase in observations with at least one occurrence of the behavior.

**Fig 2:**
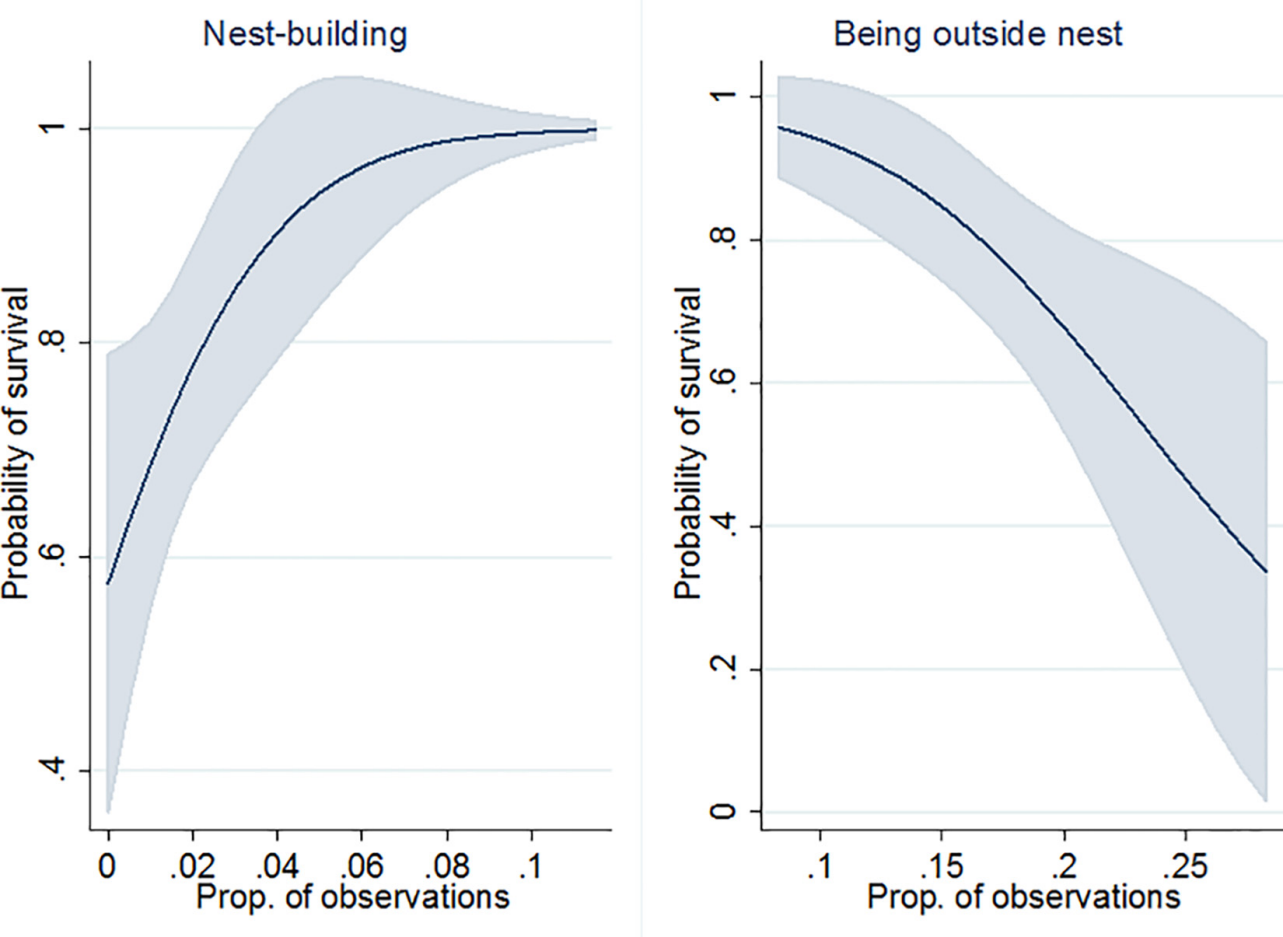
Predicted relationship between female laboratory mouse behavior and litter survival according to a multivariable logistic model. Nest building during 24 h before parturition (left) and being outside nest from 24 h before until 24 h after parturition (right) in 64 primiparous mice; behaviors expressed as mean proportion of 30-s observations with at least one occurrence of the behavior, shaded areas are 95% confidence intervals.

The final multivariable model of litter survival contained independent variables for nest building before parturition and being outside the nest during the observation period, together accounting for 33% of the variation in survival (Table 4). Because the effects of recording, cage design and mouse genotype were non-significant (p>0.25) in the first analytical step, they were not included in the third step and the final model. Predictive marginal means with 95% confidence intervals are shown in Fig 2. The probability of litter survival was predicted to increase from around 0.6 to 1 if the proportion of nest-building observations before parturition increased from 0 to 0.1, and to drop from almost 1 to 0.3 if the proportion of observations with the female being outside nest increased from 0.08 to 0.29.

**Table 4:** Final multivariable model of litter survival in 64 primiparous laboratory mice.

Independent variable^1^	Coefficient	Standard error	OR^2^	p
Intercept	4.01	1.61	-	0.013
Nest building before parturition	59.1	26.3	1.81	0.024
Being outside nest during observation period	-22.4	8.25	0.80	0.007

^1^ Proportion of 30-s observations with at least one occurrence of the behavior.

^2^ OR = change in odds of litter survival per percent unit increase in observations with at least one occurrence of the behavior.

## 4. Discussion

Loss of entire litters of newborn pups is a problem in many facilities breeding laboratory mice, with the underlying causes still poorly understood. In order to efficiently prevent litter loss, it is important to find key events relevant for survival. Proper maternal behavior is crucial for the survival of newborn mouse pups and we therefore focused on observing females just before and after giving birth.

We found that females that successfully weaned their litters performed more nest building behavior during the last 24 h before parturition. Mouse pups are born naked with no abilities to thermoregulate and without insulation from a nest and warmth from the mother mouse pups rapidly lose body temperature. It is thus of high importance for the survival of her offspring that the female prepares a nest of high quality before the pups are born. Brown [24] reported the nest condition at and shortly after parturition to be the most important factor related to offspring survival, and Gaskill *et al*. [10] found a nearly 27% increase in pup survival when providing C57BL/6 mice with enough nest material compared to raising a litter without nesting material. We found nest building before parturition to be the behavior of the female most strongly associated with litter survival.

During the first days after birth the mouse mother spends most of her time in close proximity to the pups. We found being outside nest after parturition to be the female behavior second most strongly associated with low litter survival. Furthermore, mothers performing more passive maternal behaviors, both before and after giving birth, had higher litter survival. Spending more time inside the nest and being more passive might give the pups a better opportunity to suckle and therefore increase their chances of surviving. In mice, nursing has been reported to account for 92% of the maternal behavior during the first three weeks after birth [30]. However, we also found that a moderate amount of active maternal behavior is associated with maximum survival. Being active during certain periods may be important for proper caretaking of the pups.

We also found an association between the female ignoring still pups during the first 24 h after parturition and low litter survival. Newborn pups outside the nest have limited possibilities to move back to the nest and they therefore mainly depend on the mothers retrieving ability. In a previous study [26] we found that pups displayed fewer and smaller movements before they eventually stopped moving. If the mother does not retrieve these pups back into the nest, they will rapidly lose body temperature and may die. Weak pups might not vocalise which otherwise triggers retrieval behavior in mouse mothers [31]. It might therefore be of great importance that the mother is attentive and notice pups that are lying still outside the nest.

Several studies have investigated the effect of different factors (e.g. strain, housing systems, nesting material) on maternal behavior [19, 32] and reproductive performance [16, 33–39], and differences both in terms of maternal behavior and survival of offspring have been reported. Others have studied the effects of specific induced mutations on the survival of laboratory mouse pups and poor maternal behavior has been found in several models and some even show complete inability to rear offspring (*e*.*g*. [40–42]). These studies can increase the knowledge of biological functions and give interesting insights into genes involved in reproduction. However, most of these studies are made from a perspective of understanding biological processes rather than understanding why mouse pups die, limiting the application of these results on pup mortality in breeding facilities. In cases where gene mutations lead to neonatal death, pup deaths are not always a direct consequence of the primary defect, but often caused by physiological problems that arise as secondary effects (reviewed in [43]).

This is the first study to compare the behavior of female mice that successfully weaned their litter with the behavior of females whose litters died. Malmkvist *et al*. [22] made a similar comparison in farmed mink and in line with our results they found several behavioral differences between the groups. Both the duration of parturition and birth problems were related to early kit mortality. During the first 24 h after birth, females with high mortality carried kits less and placed them less often at the udder. This is in accordance with our observation that females that lost their litters performed more parturition-related behaviors, which might indicate problems when giving birth. Indeed, in a previous paper reporting more detailed observations of these females, we found evidence of problematic parturitions in several of them, such as dystocia, prolonged parturition and lying in a hunched posture outside nest after parturition [26]. An influence of problematic parturitions on survival of offspring has also been found in pigs, with prolonged farrowing reported to increase the proportion of stillborn piglets [23].

## 5. Conclusions

Nest building before parturition, having an uncomplicated parturition, spending time inside the nest, not ignoring pups falling outside the nest and passive maternal behavior displayed by female laboratory mice were identified as important for the survival of their pups. The probability of litter survival increased dramatically with nest building behavior, and decreased with the female being outside nest. The occurrence of nest building before parturition and being outside nest together accounted for a third of the total variation in litter survival.

## Supporting Information

S1 Data All data collected from the study. (XLSX)

S1 Ethogram Overview of all the behaviors used for observing mice, description of how the behaviors were defined and the categories each behavior was included in. (DOCX)

S1 Fig Housing systems used in the study. (A) Makrolon II, corncob bedding, no nesting material, no furnishment (recording A); (B) Makrolon III, corncob bedding, 100 ml aspen bedding, half a tissue of paper, translucent red PVC nest box, cardboard nest box, chew block (recording A); (C) Makrolon II, corncob bedding, half a Nestlet, no furnishment (recording B); (D) Makrolon II, corncob bedding, one Nestlet, nest tube, a transparent tinted polycarbonate mouse tunnel (recording B).
(DOCX)
