# Correction: Do Laboratory Mouse Females that Lose Their Litters Behave Differently around Parturition?

**DOI:** 10.1371/journal.pone.0165578

**Published:** 2016-10-21

**Authors:** The PLOS ONE Staff

The following information is missing from the Funding section: Publication of this paper was funded by FEDER—Fundo Europeu de Desenvolvimento Regional funds through the COMPETE 2020—Operational Programme for Competitiveness and Internationalisation (POCI), Portugal 2020, and by Portuguese funds through FCT—Fundação para a Ciência e a Tecnologia/Ministério da Ciência, Tecnologia e Inovação in the framework of the project "Institute for Research and Innovation in Health Sciences" (POCI-01-0145-FEDER-007274). IASO received this funding. The publisher apologizes for the error.
