# Supplementary material for: Do Laboratory Mouse Females that Lose Their Litters Behave Differently around Parturition?
Source: PLoS One. 2016 Aug 30;11(8):e0161238. doi: 10.1371/journal.pone.0161238 (PMC5005013; doi:10.1371/journal.pone.0161238)
Supplement: S1 Ethogram — (DOCX) [file pone.0161238.s002.docx]

# S1 Ethogram. Overview of all the behaviors used for observing mice, description of how the behaviors were defined and the categories each behavior was included in.

| Category | Behavior | Description |
| --- | --- | --- |
| Parturition-related | Giving birth | Giving birth to pup and pup visible |
|  | In labor position | Stretching, standing on hind legs in parturition posture, moving body up and down, but no pup visible |
|  | Dystocia | Pup stuck in the genitals during parturition for an entire 30-s video recording period |
| Nest building | Nest building | Female visible and manipulating bedding or nest building material from inside the nest or towards the nest or, if no nest present, towards a specific resting site |
|  | Moving nest | Picking up nest material from the nest and moving it to another part of the cage |
| Active maternal behavior | Active in nest | Female moving inside nest (not possible to see if being active with pup, thus distinguished from Active with pup) |
|  | Being active with pup | Manipulating pup inside nest (licking, lifting or other interactions with pups), pup visible |
|  | Retrieving still pup | Picking up still pup from outside the nest and returning it to the nest; pup defined as still when no movements are detected during an entire 30-s video recording period |
|  | Retrieving moving pup | Picking up moving pup from outside the nest and returning it to the nest |
|  | Carrying pup | Carrying pup around in cage without placing it in the nest (does not include moving the pup from one location to another; see Moving pup) |
|  | Moving pup | Moving pup from one location to another, but not to nest (does not include lifting or pushing the pup to outer edge of nest or outside nest, ) |
| Passive maternal behavior | Still in nest | Being still in nest (not possible to see if lying in nursing posture) |
|  | Nursing | Lying in nursing posture with pups under belly |
| Self-oriented | Resting alone | Resting without having body contact with pup |
|  | Resting outside nest | Lying still outside nest, pups not being present (before parturition) |
|  | Ignoring moving pup | Not interacting with pup that is moving outside nest for an entire 30-s video recording period. Also includes ignoring pup that is moving while lying in wrong position (back towards female, head out from female) inside nest while the female is lying still or nursing |
|  | Ignoring still pup | Not interacting with pup that is lying still outside nest for an entire 30-s video recording period. Also includes ignoring pup that is lying still in wrong position inside nest while the female is lying still or nursing |
|  | Self-grooming | Licking own fur, grooming with the forepaws, or scratching herself with any limb |
|  | Eating | Rearing up to gnaw at food pellets through the bars of the food hopper or eating food pellets on cage floor |
|  | Hunched posture | Walking or lying inside or outside nest in a hunched posture (does not include hunched nursing posture) |
|  | Digging | Digging in bedding with movements not directed towards the nest |
|  | Stretching | Extension of the hind limbs while being outside nest |
| Abnormal | Removing pup | Pushing or lifting pup from nest to the outer edge or outside nest. Does not include when pups are pushed outside nest as a consequence of female digging inside nest |
|  | Eating pup | Interacting with pup or parts of pup with mouth, decreasing the size of the pup |
|  | Tail chasing | Chasing or catching own tail |
|  | Bar gnawing | Gnawing on cage bars |
|  | Other abnormal | Performing other abnormal or stereotypic behaviors |
|  | Cage bars | Climbing cage bars |
|  |  |  |
| Location | Outside nest | Being outside nest |
|  | In nest | Being inside nest |
