# Supplementary material for: Do Laboratory Mouse Females that Lose Their Litters Behave Differently around Parturition?
Source: PLoS One. 2016 Aug 30;11(8):e0161238. doi: 10.1371/journal.pone.0161238 (PMC5005013; doi:10.1371/journal.pone.0161238)

**S1 Fig. Housing systems used in the study.** (A) Makrolon II, corncob bedding, no nesting material, no furnishment (recording A); (B) Makrolon III, corncob bedding, 100 ml aspen bedding, half a tissue of paper, translucent red PVC nest box, cardboard nest box, chew block (recording A); (C) Makrolon II, corncob bedding, half a Nestlet, no furnishment (recording B); (D) Makrolon II, corncob bedding, one Nestlet, nest tube, a transparent tinted polycarbonate mouse tunnel (recording B).

**A**

**B**

**C**

**D**


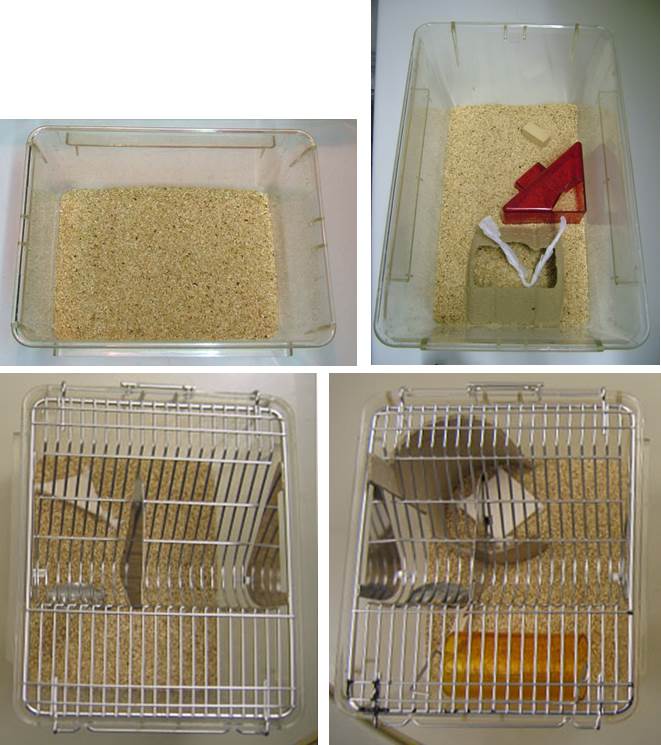

Supplement: S1 Fig — (A) Makrolon II, corncob bedding, no nesting material, no furnishment (recording A); (B) Makrolon III, corncob bedding, 100 ml aspen bedding, half a tissue of paper, translucent red PVC nest box, cardboard nest box, chew block (recording A); (C) Makrolon II, corncob bedding, half a Nestlet, no furnishment (recording B); (D) Makrolon II, corncob bedding, one Nestlet, nest tube, a transparent tinted polycarbonate mouse tunnel (recording B). (DOCX) [file pone.0161238.s003.docx]
